# Heat-related deaths in social care in England: can the Care Quality Commission ratings system be used to identify care homes that would benefit most from heat adaptation measures?

**DOI:** 10.1093/ageing/afag100

**Published:** 2026-04-27

**Authors:** Shakoor Hajat, Shereen Hussein, Keyi Li, Sari Kovats, Michael Davies, Anna Mavrogianni, Rajat Gupta

**Affiliations:** Faculty of Public Health and Policy, London School of Hygiene & Tropical Medicine, London, UK; Faculty of Public Health and Policy, London School of Hygiene & Tropical Medicine, London, UK; Faculty of Public Health and Policy, London School of Hygiene & Tropical Medicine, London, UK; Faculty of Public Health and Policy, London School of Hygiene & Tropical Medicine, London, UK; The Bartlett Faculty of the Built Environment, University College London, London, UK; The Bartlett Faculty of the Built Environment, University College London, London, UK; Oxford Institute for Sustainable Development, Oxford Brookes University, Oxford, UK

**Keywords:** climate change, heat stress, care home residents, older people

## Abstract

**Background:**

The health impacts of rising temperatures in care home settings are of growing concern. We seek to characterise the risk of heat-related mortality in nursing and residential care home settings in England and to assess potential modification of heat effects by Care Quality Commission (CQC) ratings.

**Methods:**

Heat episode analysis was used to assess excess mortality during the heatwave of 16–20 July 2022. Daily time-series regression analysis employing Distributed Lag Non-linear Models was used to assess short-term associations between daily mean temperature and daily number of deaths in care home residents during 2022–24, adjusting for season and day-of-week effects.

**Results:**

Nursing home deaths increased by 34.1% (95% CI 21.1, 48.2) and residential care home deaths by 13.0% (0.1, 27.0) during the July 2022 heatwave. During 2022–24, the relative risk of death on a day of 25°C compared to a day of 16°C was 2.09 (95% CI 1.68, 2.60) among nursing home residents and 1.56 (1.24, 1.96) in residential care homes. There was a gradient of increasing heat-related mortality risk associated with poorer CQC rating, although almost all CQC categories were associated with raised risks. Heat-related mortality risk in care homes was greatest in the West Midlands and London regions.

**Conclusions:**

Our findings indicate a growing need for heat stress to be recognised as an important risk factor for care home residents. Urgent and wide-scale improvements in heat adaptation strategies are needed in care homes across England to help improve the resilience of the social care system to climate change.

## Key Points

- There is a growing need for heat stress to be recognised as an important risk factor for care home residents.
- Our results show a gradient of increasing heat-related mortality risk associated with poorer Care Quality Commission rating.
- Urgent and wide-scale improvements in heat adaptation strategies are needed in care homes across England.

## Introduction

Climate change is increasingly recognised as a risk to health and social care delivery [1]. The health dangers associated with hot weather are of particular concern among older age groups. For example, of the almost 3000 heatwave-related deaths that occurred in England during the hot summer of 2022—including during a brief spell on 16–20 July when temperatures exceeded 40°C for the first time in the UK—over 95% of the death toll was in those aged 65 years and over [2].

Care homes are an important setting for managing risks to health and wellbeing from heatwaves [3]. Almost half a million people currently live in care homes in the UK, housed in ~16 566 facilities of which around 70% are residential care homes, i.e. providing accommodation and personal care, and around 30% are nursing homes that also provide personal care but additionally the presence of one or more qualified nurses on duty to provide nursing care [4]. The proportion of care home residents living in nursing homes is increasing and is now close to 50% of the total care home population [5]. All care homes in England are rated by the Care Quality Commission (CQC), the independent regulator of health and adult social care in England.

Care homes in the UK are generally designed to retain heat during winter to protect against cold risks, but these measures may increase the risk of summertime overheating if appropriate cooling strategies are not available [6]. Evidence of high indoor temperatures in care homes has increased in recent years. One study monitoring indoor temperatures in two London care homes found that daytime temperatures exceeded 30°C with prolonged periods of overheating, especially in bedrooms during nighttime [7]. The ClimaCare project collected longitudinal temperature data from a panel of 30 care home settings in England to characterise the risk of summertime overheating [8]. The data revealed substantial differences in indoor temperatures between care homes, reflecting variations in building design, location and heat management strategies. Since the data were collected during the summer of 2022, they also provide a unique record of monitored indoor temperatures in care homes during the record-breaking July 2022 heatwave.

The impacts of climate change and rising temperatures on the health of care home residents are, therefore, of growing concern [9]. An epidemiologic assessment of mortality data from 1993 to 2003 in England observed a higher risk of heat-related death in nursing and care home residents compared to the general population [10]. To respond to these risks, tailored heat protection guidance is now provided to social care managers and staff to implement during hot weather [11]; but it remains unclear if this has helped to reduce impacts. Also, much of the guidance focuses on short-term actions that can be taken in anticipation of and during extreme heatwave events. However, the role of the built environment in reducing heat exposure is increasingly recognised as an important adaptation strategy against rising temperatures and climate change, both at the urban and building level [12].

With a predicted need for an additional 144 000 care home beds in the UK over the next 10 years to keep pace with population growth and ageing [4], it is important to understand the impacts of hot weather on health among care home residents and to identify those settings that would benefit most from additional heat adaptation measures. This study, therefore, seeks to address the following research questions: (i) are care home residents still at higher risk of heat-related mortality compared to the wider population?; (ii) are building or management quality indicators, as reflected by CQC ratings, associated with modification of this heat-mortality risk?; and (iii) what do recent indoor temperature records reveal about environmental exposure conditions in care homes during extreme heat events?

## Methods

### Data

All-cause mortality records from England during 2022–24 were obtained from the Office for National Statistics. This period was chosen to represent current risk and includes the hot summer of 2022. Earlier years were not considered due to the impacts of COVID on mortality patterns. Daily surface-level mean temperature data were available from ERA-Land as a 0.1° × 0.1° spatial grid [13]. The dataset provides polygons in JSON format, which were matched to region names to create a composite temperature series for each Government region of England.

A full list of care homes in England and their latest ratings was obtained from the CQC directory [14]. Ratings are categorised as either ‘Outstanding’, ‘Good’, ‘Requires improvement’ or “Inadequate’. Thirty-three care homes (0.21%) were categorised as ‘not rated’ and one (0.01%) categorised as ‘Insufficient evidence to rate’ and so were excluded in the analysis by ratings. Of the five domains considered by CQC to determine ratings (Safety, Effective, Caring, Responsive and Well led), we focused on the Safety domain for our analysis as it includes consideration of safety from environmental hazards, including heatwaves [15]. We also assessed the Overall rating, which is a combination of the above five domains. The CQC also produces detailed inspection reports providing qualitative information on the quality of care for most care homes.

In addition, in order to characterise indoor temperature conditions in a sample of care homes during the July 2022 heatwave, we obtained the previously collected ClimaCare dataset [8]. These data consist of air temperatures recorded at hourly intervals using Hobo MX1101, Hobo MX1102A and Hobo MX2301 devices deployed in 30 care homes across England [16]. The monitored locations included 30 lounge areas, i.e. communal spaces such as lounges, dining rooms and lounge/diners. In addition, outdoor temperatures were monitored at hourly intervals at each of the 30 care home sites.

### Linkage

Deaths were postcode-linked to the CQC database of care homes to determine whether individuals were residents of nursing or residential care homes at the time of death. Any deaths occurring in a postcode where a care home was present were assumed to be a care home death; all other deaths were assumed to have occurred in noncare settings, described hereafter as community deaths. The average number of addresses per postcode is around 15 but can range up to 100 in some postcodes, which thus will have resulted in some misclassification error in our categorisation of care home deaths vs. deaths in the community. Deaths were then aggregated by date to create time-series of the daily number of deaths nationally and for each region of England.

### Analysis

First, excess mortality occurring during the 16–20 July 2022 heatwave was assessed using a heat episode analysis, in which the observed number of deaths during the heatwave period was compared to expected levels defined as the number of deaths occurring during the same time period in the nonheatwave years (2023–24). The percentage excess in deaths was estimated as the difference between observed and expected counts, with 95% confidence intervals (CIs) derived under the assumption of a Poisson distribution.

Next, in order to assess the general relationship between temperature and mortality rather than focusing on only a brief heatwave event, quasi-Poisson time-series regression was used to assess short-term associations between daily mean temperature and the daily number of deaths, allowing for overdispersion in the data. Natural cubic smoothing splines of time with 7 degrees of freedom per year were used to control for underlying seasonal patterns and trends in the data unrelated to temperature, along with indicator terms for potential confounding by day-of-week [17]. The seasonal control ensures that any confounding factors that change only slowly over time, e.g. demographic changes, are implicitly controlled for. We used Distributed Lag Non-linear Models employing cross-basis functions to flexibly model both nonlinear and delayed effects of temperature [18]. We considered lagged effects of up to 6 days following initial exposure since the effects of heat on mortality are known to be mostly immediate [10].

From the model, we identified the minimum mortality temperature (MMT), defined as the temperature with the lowest mortality risk, and then estimated the relative risk (RR) of death at selected temperatures compared to the MMT. Results are presented separately by CQC rating and by Government region. Further breakdown by age-group or sex was not possible due to lack of statistical power.

Lastly, the ClimaCare data were used to characterise monitored lounge and outdoor temperatures in one typical low temperature care home (defined as being in the bottom quintile of temperatures of the 30 sample care homes based on average hourly temperatures measured in care home lounges during the July 2022 heatwave) and also in one high-temperature care home (top quintile). Although there was insufficient statistical power to analyse heat-related deaths restricted to just these 30 care homes, we conducted a crude comparison of average deaths between the 15 coolest and the 15 hottest care homes in the ClimaCare dataset.

All analyses were conducted using STATA [19].

### Results

**Figure 1:**
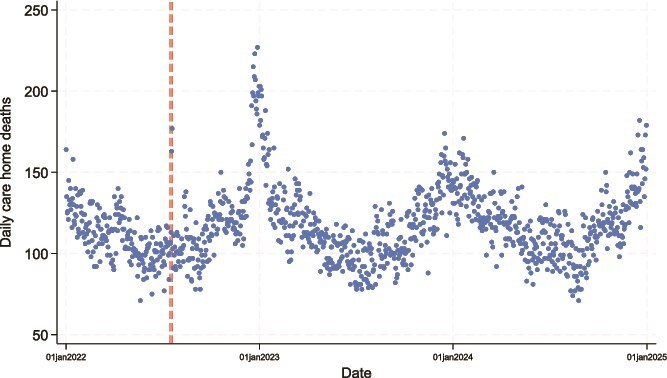
Daily care home deaths in England, 2022–24. Dashed lines indicate the heatwave period of 16–20 July 2022.

Figure 1 shows the daily number of deaths in all care home residents (i.e. both nursing and residential care homes) for the whole of England during 2022–24. The greater number of deaths observed during the winter months is typical of the mortality distribution observed in England in any regular year; however, the dashed lines indicate that during the 16–20 July 2022 heatwave period, there was a clear spike in deaths. Deaths during this 5-day period increased by 34.1% (95% CI 21.1, 48.2) in nursing homes and 13.0% (0.1, 27.0) in residential care homes. Deaths in the community (not shown) exhibited a 28.1% (25.4, 30.9) increase during the heatwave.

**Figure 2:**
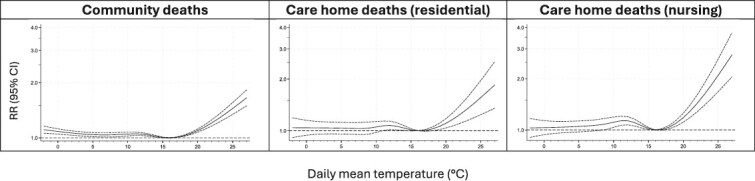
Seasonally adjusted relationship between daily mean temperature and Relative Risk (RR) of death in England by place of residence. Solid line in each figure represents the estimated risk, and the short-dashed lines either side are the 95% confidence limits.

Figure 2 shows the general relationship, after seasonal adjustment, between daily mean temperature and the RR of death for the community, residential care home and nursing home populations at the national level during 2022–24. The summed risk across lags 0–6 days is presented. The graphs show that significantly raised mortality risks are observed even at moderately high temperatures that are typically experienced most summers. Across all deaths, the value of mean temperature at which risk of death was lowest was 16°C. Comparing the three graphs, deaths among nursing home residents were most sensitive to high temperatures, i.e. exhibited the steepest slope. However, in contrast to the heatwave results, risks were greater in residential care home deaths compared to deaths in the community. The relative risk of death on a day of 25°C compared to a day of 16°C was 2.09 (95% CI 1.68, 2.60), 1.56 (1.24, 1.96) and 1.45 (1.34, 1.56) in the nursing home, residential care home and community populations respectively. Lag-specific risks (not shown) did not provide any indication of negative risk that would be consistent with a short-term mortality displacement process [20].

Table 1 shows the relative risk of death in all care home residents by CQC rating. With both the Safety and Overall domains, there is a gradient of increasing heat risk associated with successively poorer rating, although this did not reach conventional levels of statistical significance since estimates are imprecise in the ‘Outstanding’ or ‘Inadequate’ groups due to the small number of care homes rated in these categories. Homes rated either ‘Good’ or ‘Requires improvement’ were associated with significantly elevated heat mortality risk in both domains, although a raised risk was evident in almost all categories. The apparent protective effect of heat in care homes rated ‘Outstanding for Safety’ is imprecisely estimated due to the small number of homes in this category. It was not possible to run separate analysis by nursing and residential care homes due to the lack of statistical power.

**Table 1:** Relative Risk (RR) of heat-related death in all care home residents by CQC rating.

			All care home deaths
		No. of care homes	RR 25C vs. 16C (95% CI)
CQC Safety rating	Outstanding	44	0.71 (0.04, 13.43)
	Good	9986	**1.75 (1.44, 2.11)**
	Requires improvement	2506	**2.14 (1.57, 2.91)**
	Inadequate	151	2.22 (0.67, 7.29)
CQC Overall rating	Outstanding	535	1.41 (0.69, 2.91)
	Good	9789	**1.83 (1.52, 2.22)**
	Requires improvement	2236	**1.89 (1.36, 2.63)**
	Inadequate	126	3.10 (0.91, 10.63)

Results in bold are statistically significant at the 5% level.

**Figure 3:**
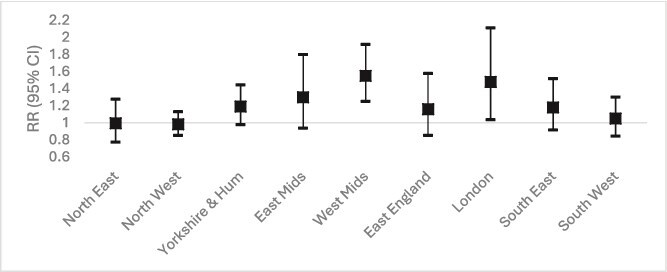
Relative Risk (RR) of care home death at the 99th vs. 50th percentile temperature, by government region.

Figure 3 shows the relative risk (RR) of heat-related death in all care home residents by government region. To allow valid geographical comparison, the RR presented is for the 99th percentile vs. the 50th percentile of the temperature distribution specific to each region. There was a raised risk in all regions except the North East and North West, although the increase was statistically significant only in the West Midlands and London. The increase in the West Midlands was over three time greater than in some other regions. The exact effect sizes are provided in Appendix 1 of the Supplementary Data section.

**Figure 4:**
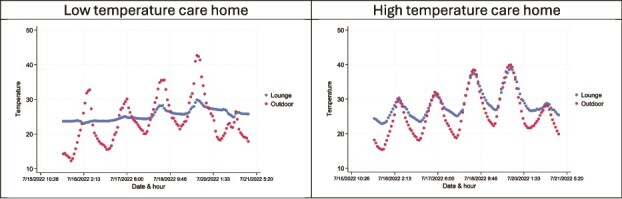
Hourly monitored lounge and outdoor temperatures (°C) in two ClimaCare care homes during the July 2022 heatwave.

Figure 4 shows hourly lounge and outdoor temperatures monitored during the July 2022 heatwave in two care homes by the ClimaCare project. Both facilities experienced similar outdoor temperatures, but the low-temperature care home had more stable lounge temperatures that were largely independent of outdoor conditions, although with some increase during the hottest days of 18 and 19 July 2022. Lounge temperatures in the high-temperature care home were much more affected by outdoor conditions. Outdoor temperatures sometimes exceeded the official Met Office record high temperature of 40.3°C due to local heat sources, e.g. reflective surfaces, creating microclimates.

Although the differential number of heat-related deaths in the 30 ClimaCare care homes were too few to robustly compare, the ratio of deaths in the coolest 15 ClimaCare care homes compared to in the hottest 15 care homes was 1:1.31 across the whole study period (2022–24) but this ratio increased to 1:2.33 in the hot month of July 2022.

## Discussion

Our findings show that heat-related deaths in care settings are a serious problem. Current risks are stronger than those reported for a previous period in England [10], indicating the increasing impact of climate change and the apparent lack of progress on climate adaptation in the care home sector.

The greatest heat risk was observed among nursing home residents, which is unsurprising given their frailty and age profile. Although the general summertime heat risk was stronger among care home residents compared to the general population, this was not the case specifically during the July 2022 heatwave. This reversal suggests that health protection measures may have been successfully implemented during this brief period in residential care home settings. Evidence from the famous 2003 France heatwave suggested that medical care directed towards the most fragile care home residents led to less frail patients contributing most to excess mortality during the heatwave [21]. Knowledge and care among German nursing home staff are also observed to be good during heat extremes [22]. The higher general heat-mortality risk in care homes in the West Midlands compared to other regions in our study is difficult to explain and is not an observation mirrored in the general population [23], although excess mortality was high in the West Midlands specifically during the 2022 heatwaves [2].

To the best of our knowledge, this study provides the first assessment of heat-related health impacts in care homes categorised by CQC ratings. Although limited by small numbers, there was modification of heat risk by CQC rating. Even homes rated ‘Good’ were associated with significantly elevated heat-related mortality risk, but the markedly higher heat risk observed in care homes rated ‘Inadequate’ is particularly concerning. In order to better understand potential contributing factors, we reviewed the detailed CQC inspection reports for those care homes rated ‘Inadequate’ for both the Safety and Overall domains. We found that none of these reports included any indication of potential vulnerability of residents to heat exposure (either outdoor or indoor) nor any mention of thermal comfort. This suggests that the modification of heat risk by CQC rating may reflect broader risk factors and underlying vulnerabilities, and so improving overall conditions in these care homes would likely help to reduce heat-related health burdens also. The explicit integration of thermal safety by CQC into its inspection domains would, however, enable better targeting of care homes that would benefit most from focused heat adaptation measures.

Some limitations of this study are acknowledged. Temperature exposure for the mortality analyses was based on outdoor measurements, which will inevitably result in some exposure misclassification. This bias is considered to lead to a flattening of the exposure–response relationship, which may result in lower effect estimates [24]. The modelling of temperature at a building level could help to improve indoor exposure information in future studies. Future work could also collect information on the prevalence of air conditioning (AC) in care homes and whether heat-mortality risk is lower in those homes that have AC access. Another limitation is that the linkage of mortality records to care home addresses at postcode level will have led to some misclassification of residents; however, any resulting bias is likely to be nondifferential. Also, although the latest CQC ratings were used, the most common year of publication of inspection reports was 2019, meaning that there is scope for the quality to have changed by the time of the study period of 2022–24, but again, this would only serve to bias results towards the null. We did not control for air pollution as a possible confounder in our regression models since exposure at the regional level would have been highly heterogeneous. The theoretical basis for air pollution control in climate epidemiology studies has, in any case, been questioned [25]. Due to power limitations, we could not consider cause-specific mortality, which limits the implications of the findings for clinical intervention and preventative actions during hot weather [26]. Finally, there are likely to be important heat-related impacts on morbidity outcomes as well, which are not quantified in this study, although a previous study from Belgium observed elevated mortality among nursing home residents during heatwaves but no increase in hospitalisations [27].

Our findings indicate a growing need for heat stress to be recognised as an important risk factor for care home residents. Although not addressed in our study, the health and wellbeing of the ~750 000 UK care home workforce during hot weather is also an important consideration. Government advice, in the form of the Adverse Weather and Health Plan, Heat Health Alert Action Cards and other documents, are available to social care providers to help minimise impacts when an extreme heat event is forecast. Recommended measures include acute actions such as patients and staff keeping hydrated, relocating to cool areas, reviewing storage of medications and staff contacting clinical leads if they have concerns regarding the health of a patient [28]. Monitoring body temperature of high-risk patients for signs of heat exhaustion or heat stroke, heart and breathing rates, blood pressure and hydration levels is also advised, as well as weighing patients regularly to identify dehydration and rescheduling physiotherapy to cooler hours. However, given that deaths in the 15 hottest ClimaCare care homes were particularly high in the hot month of July 2022 and that care home deaths in general have remained significantly elevated during recent heat episodes in England [29], more needs to be done. Routinely monitoring indoor temperatures in care homes and in different rooms (bedrooms, lounge, etc.) can help to identify heat vulnerable locations, and the CQC could play a stronger role in regulating such practices. In addition, passive cooling solutions such as increasing external shading, nighttime secure ventilation and using high-albedo materials on external building surfaces can help to lower indoor temperatures, which, in turn, can contribute greatly to reducing the health burdens associated with the nonextreme periods of hot weather when acute actions are less likely to be implemented [7]. Also, given the substantial number of frail and vulnerable individuals receiving care at home and the recent policy shift by the UK Government towards promoting more supported living as part of its 10 Year Health Plan for England [30], it is important that vulnerable individuals have access to specialist care at home when needed and are supported to keep their own homes cool.

In conclusion, our results provide evidence that heat-related mortality risk in care homes remains high. Given that significant impacts were apparent in almost all care home categories regardless of rating, there is a need for urgent and wide-scale improvements in heat adaptation strategies to improve the resilience of the social care system to climate change.

## Supplementary Material

aa-25-3393-File002_afag100
