# Supplementary material for: Heat-related deaths in social care in England: can the Care Quality Commission ratings system be used to identify care homes that would benefit most from heat adaptation measures?
Source: Age Ageing. 2026 Apr 27;55(4):afag100. doi: 10.1093/ageing/afag100 (PMC13110879; doi:10.1093/ageing/afag100)
Supplement: aa-25-3393-File002_afag100 [file aa-25-3393-file002_afag100.docx]

**Heat-related deaths in social care in England: can the Care Quality Commission (CQC) ratings system be used to identify care homes that would benefit most from heat adaptation measures?**

Supplementary Data: Appendix 1

| **Region** | **50^th^ percentile temperature** | **99^th^ percentile**  **temperature** | **Relative**  **risk** | **Lower confidence limit** | **Upper confidence limit** |
| --- | --- | --- | --- | --- | --- |
| North East | 9.23 | 18.76 | 0.994265 | 0.774356 | 1.276624 |
| North West | 9.75 | 19.33 | 0.98184 | 0.853066 | 1.130052 |
| Yorkshire & Humberside | 10.06 | 20.05 | 1.18914 | 0.9786 | 1.444977 |
| East Midlands | 10.77 | 22.04 | 1.29834 | 0.936852 | 1.799309 |
| West Midlands | 10.6 | 21.57 | 1.54952 | 1.252378 | 1.917162 |
| East England | 11.25 | 22.62 | 1.159606 | 0.852707 | 1.576961 |
| London | 11.51 | 22.85 | 1.479145 | 1.035869 | 2.11211 |
| South East | 11.49 | 22.16 | 1.179409 | 0.916744 | 1.517331 |
| South West | 11.37 | 21.12 | 1.048811 | 0.845068 | 1.301676 |
